# Supplementary material for: Unveiling a Novel Role of Cdc42 in Pyruvate Metabolism Pathway to Mediate Insecticidal Activity of Beauveria bassiana
Source: J Fungi (Basel). 2022 Apr 12;8(4):394. doi: 10.3390/jof8040394 (PMC9031566; doi:10.3390/jof8040394)
Supplement: Supplementary file 1 [file jof-08-00394-s001.zip › jof-1672492-supplementary.pdf]

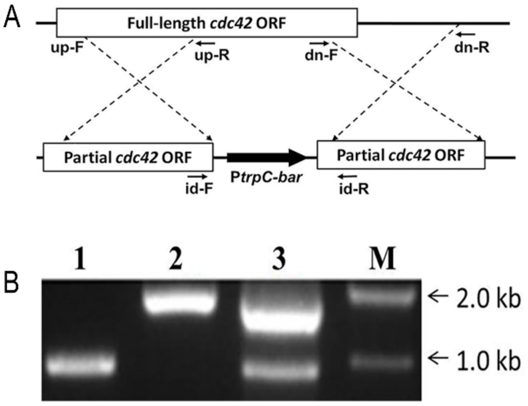

Figure S1. Construction of the *cdc42* mutants. (**A**) The strategy for *cdc42* deletion. (**B**) Gel electrophoresis for the identification of deletion and complementary strains. Lane 1: WT, lane 2: Δ*cdc42*; lane 3: Δ*cdc42::cdc42*, M: marker.

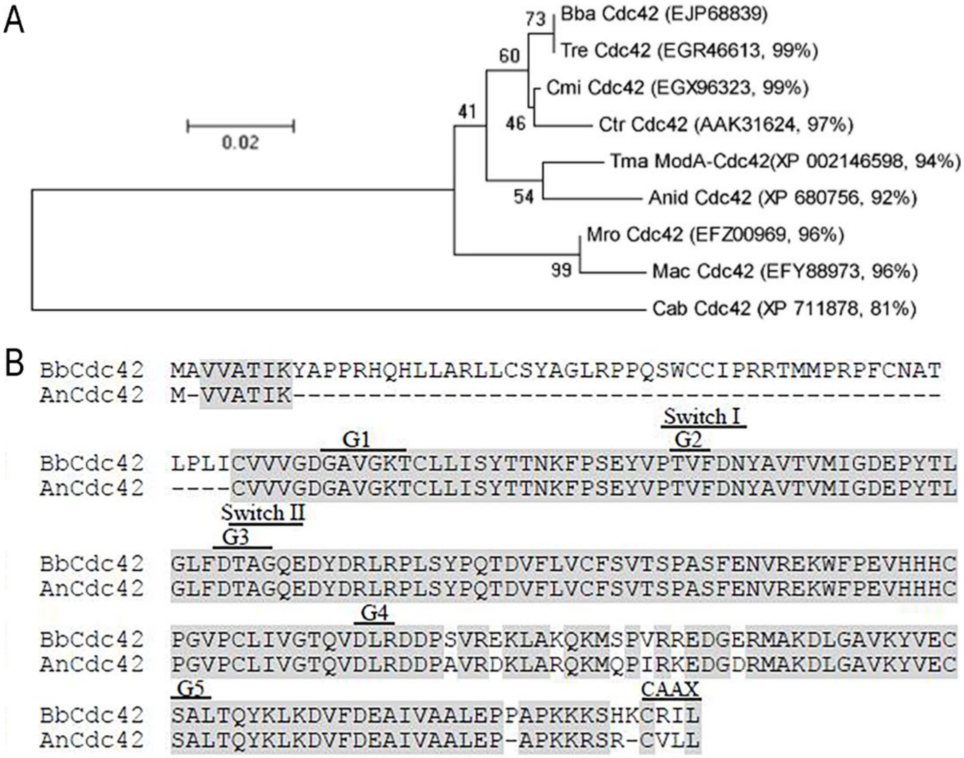

Figure S2. Phylogenetic analysis and the structural features of the Cdc42 in *B. bassiana* compared with several homologues. (**A**) Phylogenetic tree for Cdc42 homologues (NCBI codes and the identity in parentheses) in several fungi [*Beauveria bassiana* (Bba), *Trichoderma reesei* (Tre), *Cordyceps militaris* (Cmi), *Colletotrichum trifolii* (Ctr), *Talaromyces marneffei* (Tma), *Aspergillus nidulans* (Anid), *Metarhizium robertsii* (Mro), *Metarhizium acridum* (Mac), *Candida albicans* (Cab)]. (**B**) Sequence comparison of Cdc42 with the orthologue in *Aspergillus nidulans* (Anid).

Table S1. Primers for constructing gene deletion or complementary strains.

Primers	Sequences	Purpose
Cdc42-up-F/R	TCCCCCCGGGTCTTTCTGCCGAGTGAATGCC/ CGCGGATCCCGAAGACCAGCATACGAGCATAG	Cloning cdc42-5′
Cdc42-dn-F/R	AAGCTCTAGATCAGATGCGTTGTTGTCGGAGAC/ GGACTAGTTAAATGCCAGGTGAAACAGG	Cloning cdc42-3′
Cdc42-com-F/R	GGGGACAAGTTTGTACAAAAAA-GCAGGCTGCGATGTACGGATATAGATAGGCTG/GGG-GACCACTTTGTACAAGAAAGCTGGGTCGGGGG-TATGATTGATTACAAAATG	Cloning full-length cdc42
Cdc42-id-F/R	CCCCAGTCAACCTAATCACCAAATC/ GTAGAACAA-GCCGTTAGCGTCATT	Identification of gene-deletion clones
